# Finite Element Simplifications and Simulation Reliability in Single Point Incremental Forming

**DOI:** 10.3390/ma15103707

**Published:** 2022-05-22

**Authors:** Tomaž Pepelnjak, Luka Sevšek, Ognjan Lužanin, Mladomir Milutinović

**Affiliations:** 1 Department of Manufacturing Technologies and Systems, Faculty of Mechanical Engineering, University of Ljubljana, Askerčeva 6, SI-1000 Ljubljana, Slovenia; luka.sevsek@fs.uni-lj.si; 2 Department for Production Engineering, Faculty of Technical Sciences, University of Novi Sad, Trg Dositeja Obradovića 6, 21000 Novi Sad, Serbia; luzanin@uns.ac.rs

**Keywords:** single point incremental forming, numerical simulation, mass and time scaling, pillow effect, artificial neural network

## Abstract

Single point incremental forming (SPIF) is one of the most promising technologies for the manufacturing of sheet metal prototypes and parts in small quantities. Similar to other forming processes, the design of the SPIF process is a demanding task. Nowadays, the design process is usually performed using numerical simulations and virtual models. The modelling of the SPIF process faces several challenges, including extremely long computational times caused by long tool paths and the complexity of the problem. Path determination is also a demanding task. This paper presents a finite element (FE) analysis of an incrementally formed truncated pyramid compared to experimental validation. Focus was placed on a possible simplification of the FE process modelling and its impact on the reliability of the results obtained, especially on the geometric accuracy of the part and bottom pillowing effect. The FE modelling of SPIF process was performed with the software ABAQUS, while the experiment was performed on a conventional milling machine. Low-carbon steel DC04 was used. The results confirm that by implementing mass scaling and/or time scaling, the required calculation time can be significantly reduced without substantially affecting the pillowing accuracy. An innovative artificial neural network (ANN) approach was selected to find the optimal values of mesh size and mass scaling in term of minimal bottom pillowing error. However, care should be taken when increasing the element size, as it has a significant impact on the pillow effect at the bottom of the formed part. In the range of selected mass scaling and element size, the smallest geometrical error regarding the experimental part was obtained by mass scaling of 19.01 and tool velocity of 16.49 m/s at the mesh size of 1 × 1 mm. The obtained results enable significant reduction of the computational time and can be applied in the future for other incrementally formed shapes as well.

## 1. Introduction

It is well-known that the conventional methods of sheet metal processing are cost-effective only in the case of mass production and that the process effectiveness is very sensitive to the complexity of part geometry. However, the introduction of flexible, innovative, and rapid manufacturing methods such as incremental forming (IF) enabled the economical production of sheet components in small batches, prototypes, and ‘custom-made’ products; such methods appeared as early as in 2004, as reported by Esmaeilpour [1]. As Li et al. presented in 2017 [2], the SPIF technology was primarily developed for the needs of the automotive and aerospace industries, but over time, application area expanded considerably to other disciplines for which ‘custom-made’ products are sought. Ambrogio et al. [3] report this in cases from medicine and Milutinović et al. [4] in case studies from dentistry. Since the process is rather distinct in comparison to other processes of sheet metal forming, special design rules were presented by Afonso et al. [5]. The process was first used solely for the production of metal parts [6,7,8], but in recent years, various polymer materials have also been successfully processed by IF technologies [9].

In an SPIF process, a universal computer-driven punch is used for gradual (incremental) shaping of a sheet blank, which is clamped onto a frame to prevent its sliding. Duflou et al. [6] present several process variations for which the IF can be performed on a CNC milling machine or by a robot arm. However, in all cases, the proper lubrication of the contact surface is necessary. The lubrication process is influenced by several parameters being studied by Petek et al. [10]. Thanks to the dieless concept, Pepelnjak et al. [11] have pointed out high flexibility combined with low tooling costs of the SPIF process, since the tool costs, as well as the setup time, are reduced significantly in comparison to the regular forming processes. Furthermore, due to the highly localised zone of plastic strain during the forming process, the material formability is higher and forming load is considerably smaller compared to conventional sheet technologies such as deep drawing. Additionally, in contrast to conventional sheet forming, the forming forces are independent of the workpiece size [12,13]. The main disadvantages of the SPIF technology are lower dimensional and shape accuracy as well as significantly longer processing times in comparison to other sheet-metal-forming processes [14,15].

Programming of the punch trajectory and the design of a SPIF process is a very demanding task with several influential process variables (sheet thickness, punch diameter, punch velocity, step down, forming angle, etc.) and different phenomena (elastic springback, sheet bending, sheet thinning, ‘pillow effect’) that are to be considered in the planning of the tool path [16]. Tool trajectory is usually generated using commercial software for computer-aided manufacturing (CAM) being developed for a milling process. However, due to differences between milling and forming processes and due to the non-homogeneous sheet metal material, the obtained results may not be reliable enough. Therefore, special programs for the determination as well as optimisation of forming paths at SPIF are developed [17,18]. In order to consider the main phenomena of incremental sheet metal forming prior to the experimental work, the numerical analyses are indispensable. Through these digital analyses performed commonly by finite element method (FEM) simulations, the accuracy of the produced part and forming forces applied to the CNC milling machine can be predicted. That fact is indispensable when the CNC machine, which is not dedicated to forming but to specific milling operations, needs to be used.

The FEM simulations of metal-processing technologies are often implemented in industrial practice prior to the part production [19,20,21,22]. However, for process optimisation, they are often not effective enough due to long computational time, modelling complexity, and insufficient accuracy of the simulated part [18,19]. Although the majority of the simulations of sheet metal incremental forming apply shell elements, long computational times are in several cases referred to as a fundamental problem. The long computational time is a result of complex and long tool paths and fine meshing (discretisation) of the FE model, which is required due to the small and fast-changing contact zone between the forming tool and the blank [23]. In order to perform the simulations in the shortest possible time, the explicit dynamic types of solvers are mostly employed for FE simulations of the SPIF. With this, shorter computing times in comparison to the implicitly based solvers can be attained [20]. Furthermore, to additionally shorten the computational time, several approaches based on different simplifications in the process modelling have been suggested. It should be noted that some of the commercial FE programs are not able to simulate ISF processes due to the complex path of the tool [24].

As an alternative to classical flow rule theory, which is dominantly used in simulations of the metal-forming processes, Robert et al. [25] proposed a simple elasto-plastic model of material behaviour based on an incremental deformation theory of plasticity. They reported a shortening of CPU time while still maintaining a sufficient level of accuracy. Later, Robert et al. [26] developed a simplified model for the forming tool/blank contact conditions incorporated into the ABAQUS software. The new algorithm enabled shortening of the computational time by 65% compared to a standard contact algorithm. In contrast, Hadoush and van den Boogaard [27] utilised a mesh refinement/derefinement (RD) approach in SPIF simulation to overcome the necessity of having an initially fine mesh. As a result, the computing time was reduced by 50% in relation to the reference model. Muresan et al. [28] and Sebastiani et al. [29] were among the first to introduce a decoupling method for ISF process modelling in order to reduce the computational time. Starting from the fact that forming zone is localised and small compared to the rest of the blank, they subdivided the FE model into an elastic zone and an elastic-plastic deformation zone. Those two separate domains are then alternately solved in a step-wise algorithm. A similar approach was exploited by Hadoush et al. [30], who implemented a direct sub-structuring method, speeding up the SPIF simulation by a factor of 2.4 compared to the traditional implicit simulation. Hadoush et al. also combined adaptive mesh refinement with the two-domain method to further reduce the computational time. Bambach [31] reached significant saving in the CPU time (up to 80%) and satisfactory geometrical accuracy in the ISF simulation of a cone shape by combining an adaptive remeshing strategy based on a multi-mesh method and subcycling (use of different time steps for elements of different size). In his study, Bambach also provided a simple model for the CPU time calculation in a case of explicit FE simulations of the ISF process. By assuming that the CPU time per element and increment is a constant that depends only on the speed of the computer, the total CPU time can be determined from the following expression:

(1) $$t_{\mathrm{CPU}}=\frac{tool\;path\;length\;\cdot number\;of\;increments}{tool\;velocity\cdot stable\;time\;increment},$$

The possibilities of the computational efficiency improvement in the FE simulation of ISF processes using a selective element fission method are presented in [19]. The computational performance was upgraded up to 74% by dividing the toolpath into a number of segments and meshing the FE model with elements of different size in accordance with the predefined split toolpath segments and deformation regions. In that manner, the number of elements in the FE model was reduced along with unnecessary, time-consuming calculations for the elements out of the localised deformation zone. In another study dealing with shortening of the ISF simulation time, Sena et al. [32] applied an adaptive remeshing technique that automatically refines only a portion of the sheet mesh in the vicinity of the tool. In engineering practice, scaling of process time (tool velocity) and/or mass is often employed in FE explicit simulations of the ISF process (especially in case of large-size components) to overcome the problem of long computational time. This approach could be very effective in reducing the CPU time, but special attention is required regarding the definition of the tool trajectory and the selection of the scaling factors [31]. Generally, by increasing the tool velocity and the mass scaling factor, the reliability of FE model decreases, and, in particular, a noticeable inaccuracy in the shape of the workpiece and sheet thickness may occur [32]. Therefore, the scaling level must be carefully adopted, and a compromise between the simulation accuracy and the simulation time is necessary to achieve an optimal solution.

To increase modelling process accuracy and efficiency, the FE approach has been widely combined with artificial neural networks (ANN) in recent years. ANN is a promising and sophisticated computer modelling technique that can be used to simulate and optimize a variety of manufacturing processes, including metal-forming processes and multi-response parameters. The early works applying the approach of ANN combined with Taguchi method were reported already in 1999 by Ko et al. [33], where they presented the implementation of this new approach combined with FEM in the cold heading process. In 1998, Forcellese et al. [34] evaluated the effect of the training set size of ANN on the reliability of the prediction of the springback in the free-bending process, which was also presented in the overview work by Pattanaik in 2013 [35]. In the following years, the ANN methods were further developed and applied to several forming technologies including deep drawing [36], ring rolling [37], electrohydraulic forming [38], bending [39,40], incremental forming [41], and several other application areas [42,43,44,45,46]. Hamouche et al. [47] have developed a novel approach to select and classify a sheet metal process by machine-learning method from the final part geometry and achieved an accuracy of 89%. Besides the evaluation of the forming applications, other aspects influencing the forming processes were also observed. Merayo et al. have evaluated by ANN the material properties of formable materials [48,49], while Trzepieciński et al. [50] have focused on the analyses of friction coefficient evaluated by multi-layer artificial neural networks (ANNs) and backward elimination regression.

In recent years, the methods of machine learning and different ANN approaches have also been implemented in the optimizations of incremental forming technology. These methods have been particularly useful where authors have observed multi-objective optimisations [51] or sought the optimal tool path generation and manufacturing strategies [52,53]. The multi-objective optimization was also implemented in incremental forming by Taherkhani et al. [54] to achieve the best possible dimensional accuracy and surface quality in the shortest possible processing time. However, none of the researchers have focused on the optimization of the simulation of incremental forming as a function of the mesh size and mass scale factor.

The key factor in implementing the simplification of the FE model in the ISP process simulation is to know what impact the particular simplification will have on the simulation results. The present paper deals with the effects of the above-mentioned simplifications (tool velocity and mass scaling) in combination with the finite element size (mesh density) on the FE modelling results in the case of a classic SPIF process. In order to shorten the computational times of the simulations, our objective was to develop an utterly and completely simplified FE model with sufficient geometrical accuracy. The FE model created in the ABAQUS commercial software package was verified by comparing the predicted digital geometry of the workpiece (truncated pyramid) with the real one (experimentally obtained). Part accuracy and in particular the ‘pillow effect’ are discussed as well.

## 2. Accuracy of the SPIF Process

The accurate prediction of the geometry of the final part (i.e., the expected part dimensional and shape errors) is essential for the proper design of the SPIF process. However, SPIF numerical modelling is a very challenging issue, since the geometric accuracy of the workpiece in the SPIF process is influenced by many factors: process variables, tool and workpiece geometry, sheet thickness, mechanical properties of the material, friction, design and stiffness of clamping system, etc.

Geometric errors of the final parts obtained by the SPIF can be divided into three different categories [15]. At the very beginning of the SPIF process, an undesirable bending of the clamped sheet occurs along the main base of the workpiece, resulting in discrepancies between the actual part geometry and the desired one. This is caused by the vertical component of the forming load acting at the selected distance from the blank holder, which results in the fact that the blank is prone to bending rather than being only locally deformed in the vicinity of the forming tool. The sheet bending effect is especially noticeable in the case of a thin sheet and/or poor support of the sheet blank. Using a simple backing plate or applying the rigid support next to the forming zone limits the sheet’s undesired bending and, consequently, related geometric errors.

Another source of part inaccuracy is the well-known phenomenon of material elastic springback caused by a sudden drop in the stress when the formed part is unloaded. In the SPIF process, due to the gradual manner of the part’s plastic deformation, springback occurs not only at the end of the forming process but also locally during the entire process. In other words, as the tool moves from point to point, there is a continuous local springback around the tool affecting the deviation in the wall geometry. Global springback that occurs after the tool is released causes sheet/workpiece lifting, and hence, the actual depth of workpiece is lower than the desired one. Elastic springback may also occur after the release of the workpiece from the clamping device and after the trimming operation. Since the amplitude of this springback is influenced by the part shape, sheet thickness, and material properties of the sheet metal, the geometrical errors should be considered for each part separately [55]. Moreover, cyclic loading and unloading of the workpiece during the SPIF process also increase the residual stresses, which amplify the springback effect and overall geometric inaccuracy. In addition to the careful selection of the process parameters, elastic springback can be minimised by global or local heating of the workpiece before and/or during the process [20].

**Figure 1:**
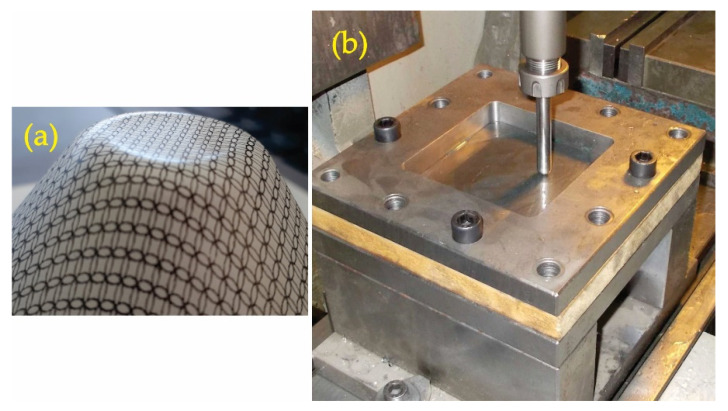
Bottom pillowing (**a**) (University of Ljubljana) and (**b**) tool-set (University of Novi Sad).

The third type of geometric error in the SPIF is a protrusion or concave curvature occurring on the undeformed bottom of the part. This phenomenon is known as a ‘pillow effect’, and according to Isidore et al. [56], it is one of the main reasons for geometric inaccuracy in the SPIF process. The pillow effect is a result of sheet bulging due to the transition of elastic deformations from the non-plastically deformed central zone to the rest of the workpiece [57] as shown in Figure 1, but some unknowns regarding this phenomenon exist, making it difficult to predict and control. This phenomenon is particularly emphasised on parts with a flat bottom section.

## 3. Materials and Methods

**Figure 2:**
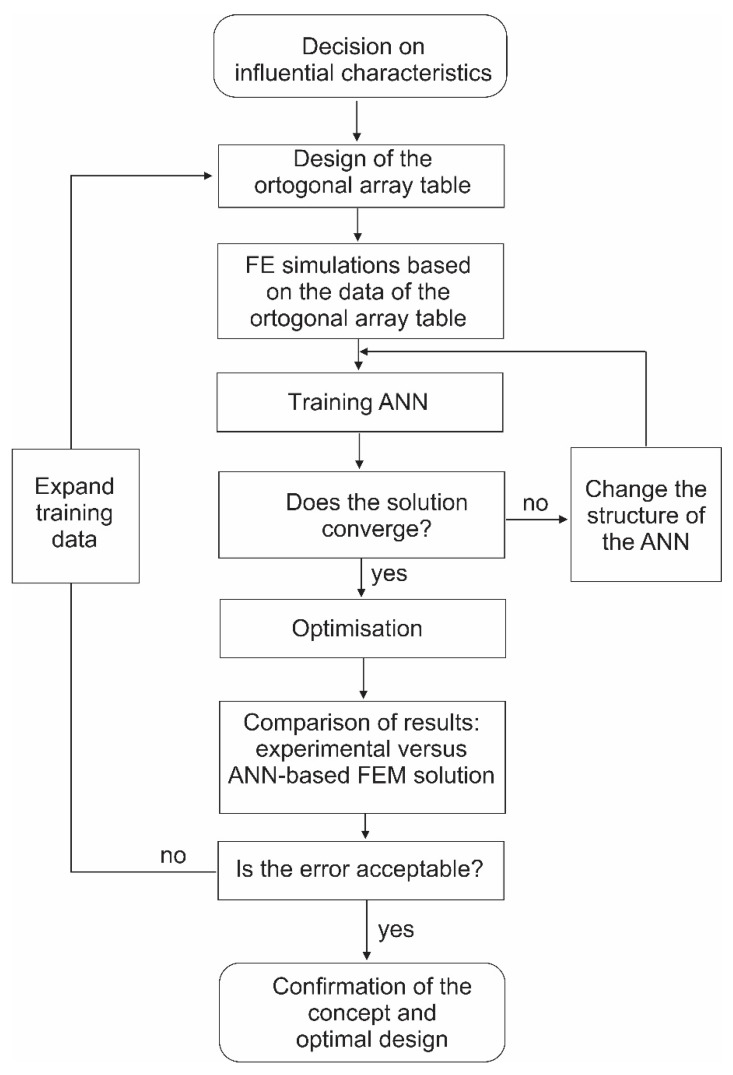
Flow chart of the performed research work.

In the presented work, the influential parameters on bottom pillowing of an incrementally-formed sheet metal part were analysed. According to the selected parameters of interest, the orthogonal array matrix was defined. This matrix represents the basis for the parameter values used in the FEM simulations. Based on the results of the FE simulations, the training of the ANN was used to specify the optimal parameters of the defined parameter design space. The quality of the ANN was finally proven by numerical simulation with optimal values of the mass scaling and mesh size parameters and its comparison with the measured experimental part (Figure 2).

### 3.1. Experimental Setup

The SPIF process was performed on a conventional CNC vertical milling centre (HAAS TM1) using a steady frame, which was fixed to a machine table (Figure 1b).

**Figure 3:**
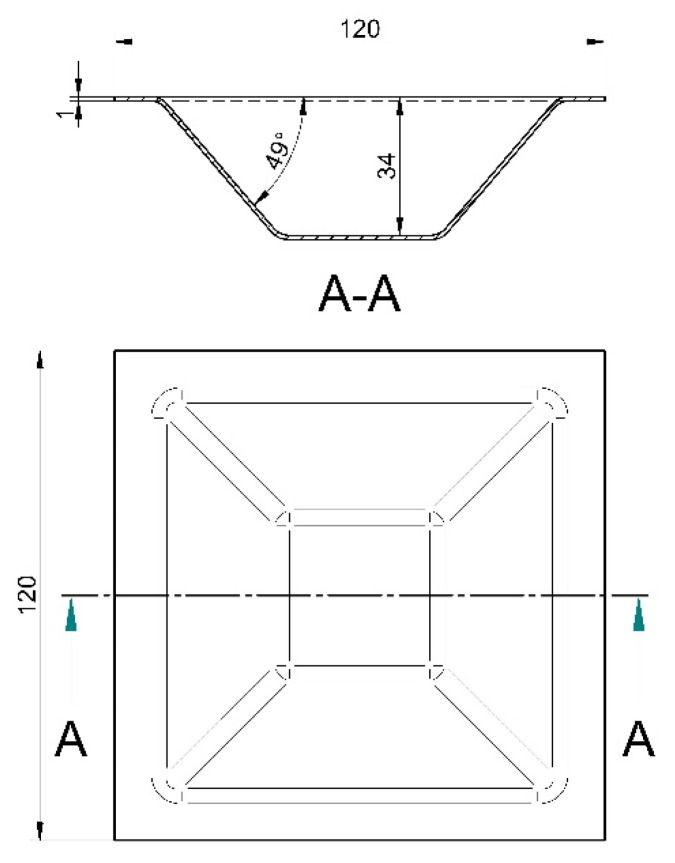
Geometry of a workpiece.

A punch with a semi-spherical head (Ø10 × 100 mm) made from tool steel EN X210Cr12 was used. It was hardened to 60HRC, ground finely, and polished. The shape and preferred dimensions of the workpiece are given in Figure 3. A square sheet metal plate (120 × 120 × 1 mm) made from low-carbon steel quality DC04 according to EN 10130 standard was used as a blank and clamped into the steady tooling frame. Both the punch and the surface of the formed workpiece were lubricated with conventional mineral oil. All the process parameters were carefully selected and optimised through a couple of tests to obtain the geometry of the workpiece with minimal deviations from the CAD model presented in Figure 3.

**Figure 4:**
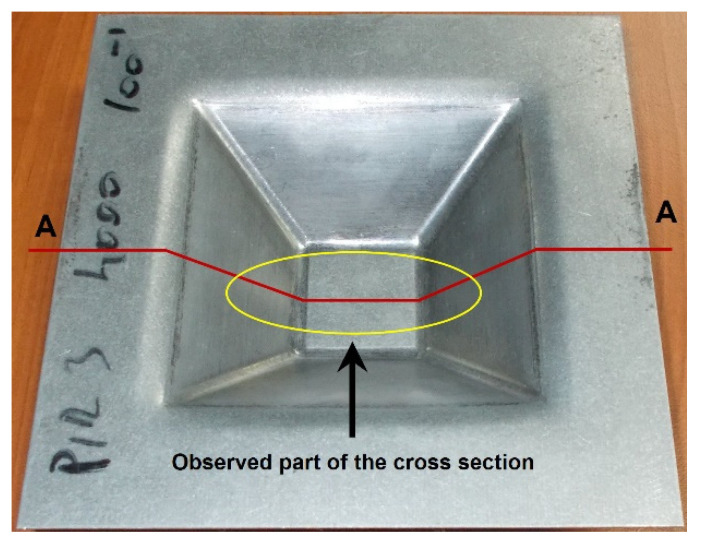
Incrementally shaped workpiece and position of the analysed cross-section.

The forming strategy (tool trajectory) was programmed as ‘profile milling’ or 2½ D contour path. The tool path and NC code for the CNC milling machine were generated using the EdgeCAM CAD/CAM software. The same tool path as for the experiment was also applied in the FEM simulation. The feed rate of the tool was 0.04 m/s with a rotating speed of 1000 rpm and vertical pitch (*Δz*) of 0.5 mm at each change of the contour path. The total forming depth was 34 mm, with a wall angle of 49°. The profile of the incrementally formed truncated pyramid (Figure 4) was scanned using a 3D coordinate measuring machine (Carl Zeiss Contura G2). The acquired geometrical data or ‘cloud of points’ were processed and converted to an STL model that can be used in conventional CAD software. The scanned geometry of the workpiece was subsequently compared along the cross-section A-A (Figure 4) with the FE predicted geometry.

### 3.2. Finite Element Analysis

The best way to better understand SPIF is through FE analysis. It provides for individual quantitative investigations of different process parameters, such as stress state, thickness, and geometry, in every observed point of the mesh. In the present study, ABAQUS/Explicit was used to simulate the investigated SPIF process. The FE model contained a hemispherical ball end tool and a backing plate that were modelled as discrete rigid 3D bodies. The toolpath in the simulations was generated directly from the CAM model and was identical to the one used in the experimental production of the test part.

In order to determine the toolpath in the ABAQUS program, it is necessary to know every coordinate of the points where the tool changes its direction. The software calculates a path between two consecutive data points by linear interpolation. In order to shorten the computational time, the tool speed was virtually increased. In the first case, the tool speed of 10 m/s was selected, while in the second case, the tool speed was increased to 40 m/s. The blank was modelled as a 120 × 120 mm elastic-plastic deformable shell with a flow curve: *σ*_f_ = 209 + 542.8·*ε*^0.67^ in MPa. It was meshed with quadrilateral structured shell elements of the S4R type. The shell elements were determined with five integration points on the sheet metal thickness. Since the size of the mesh has a crucial effect on the simulation results, its effects were investigated. Two elements having the sizes of 3 × 3 mm and 1 × 1 mm were selected. All translations and rotations were constrained for the nodes along the four edges of the blank. The Coulomb’s friction model with a friction coefficient of *μ* = 0.1 for lubrication with the mineral oil was selected for the tool–workpiece contact. The simulation consists of two steps aiming at SPIF forming in the first step and removing of the tool–specimen contact in the second step. Since ABAQUS/Explicit does not deliver reliable results of the springback phenomenon, this step was performed with ABAQUS/Standard implicit solver.

Finally, the influence of mass scaling or strain rate on material properties was analysed as one of the commonly used parameters in explicit simulations involving long tool paths [55]. Most studies of the SPIF process, in which density manipulation (increase) was used, select the highest value of mass scaling of 10 [55].

However, due to the long processing times of the SPIF, FE simulations using higher mass scaling factors were also first evaluated in the study presented here. Since the simulated part accuracy is of the highest importance, the optimal combination of mass scaling and tool velocity was sought. For this purpose, combined FEM simulations and design of experiment were used. The work was done stepwise to define first the separate influences of mass scaling and element size, while the tool velocity range was defined within an initial research work. Finally, the combined influences were evaluated in order to minimise the error estimation of the pillowing effect on incrementally produced test part.

### 3.3. Design of Experiment

Following the goal of the numerical prediction of the incrementally formed specimen by FEM, the presented parameters of mass scaling, tool velocity, and element size were evaluated by the statistical design of experiment approach combined with neural network. Neural networks are employed in many fields as efficient tools for modelling and optimization [58,59]. The basic idea was to train a neural network based on the data from a space-filling design, i.e., Fast and Flexible Filling Design, which is the option to use in cases where one or more factors are categorical. The total number of 30 design points was split between the two levels of the categorical factor, mesh size, so that on each categorical level there were 15 design points. Generally, such a number of points is small for serious neural network training purposes. However, JMP’s Fast and Flexible Filling Design algorithm generates those points as the cluster centroids of a much larger number of points, generated to fill the design space that is defined by the two continuous variables, tool speed and mass scale. This significantly reduced the number of design points and, subsequently, the required FEM experiments, while still supporting the quality of the neural network training. JMP software uses the MaxPro criterion [60] to maximize the product of the distances between potential design points, so that all factors are involved, thus providing good space-filling.

If *p* is the number of factors and *n* the specified number of experimental runs, the MaxPro algorithm finds cluster centroids that minimize the following criterion:

(2) $$C_{\mathrm{MaxPro}}=\sum_{i}^{n-1}\sum_{j=i+1}^{n}\left[\frac{1}{\prod_{k=1}^{p}{\left(x_{ik}-x_{jk}\right)}^{2}}\right]\;\;,$$

Once the 30 design points were defined, the design table was generated, containing 30 different settings based on which 30 FEM experiments were run (15 at each mesh size level).

### 3.4. Neural Network Architecture

**Figure 5:**
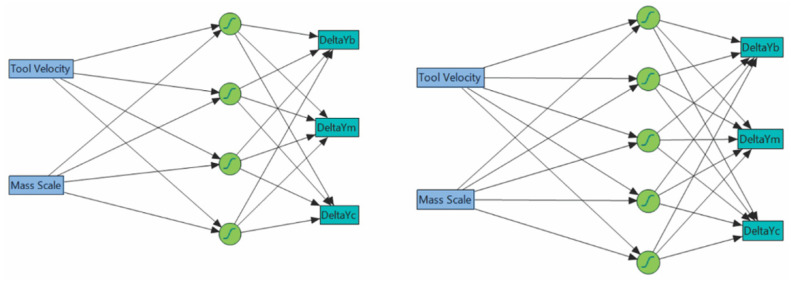
Neural network architectures for mesh size 1 × 1 mm (**left**), and mesh size 3 × 3 mm (**right**).

Typically, the task of neural network is to fit high-dimensional models. Although in our case multidimensionality was not a key issue, the task was the same, and that was to define a set of matrix parameters and then modify them to stabilize the error, i.e., to minimize the error between the sample value and the actual value. The JMP software and similar statistical modelling software allow the designer of neural network to define the number of hidden layers and the number of nodes in each layer. Most often, one hidden layer is enough for the network level, while the number of nodes usually starts from three and can be increased if necessary. In this experiment, standard architectures with 4 and 5 hidden nodes were used for mesh size 1 × 1 mm and 3 × 3 mm, respectively (Figure 5). TanH activation function was used for all network nodes. The training was based on *k*-fold cross-validation, best suited for small training sets. The observations were partitioned into 5 folds. During each iteration, the model was fit using the observations not in the current fold. The log-likelihood based on that model was computed for the observations which are in the current fold. Thus obtained, this log-likelihood value was used for validation. Once the mean of the validation log-likelihoods for the *k* folds were calculated, the resulting value was used as a validation log-likelihood for the tuning parameter. Based on this process, the tuning parameter with the maximum validation log-likelihood was used to construct the final solution.

## 4. Results and Discussion

**Figure 6:**
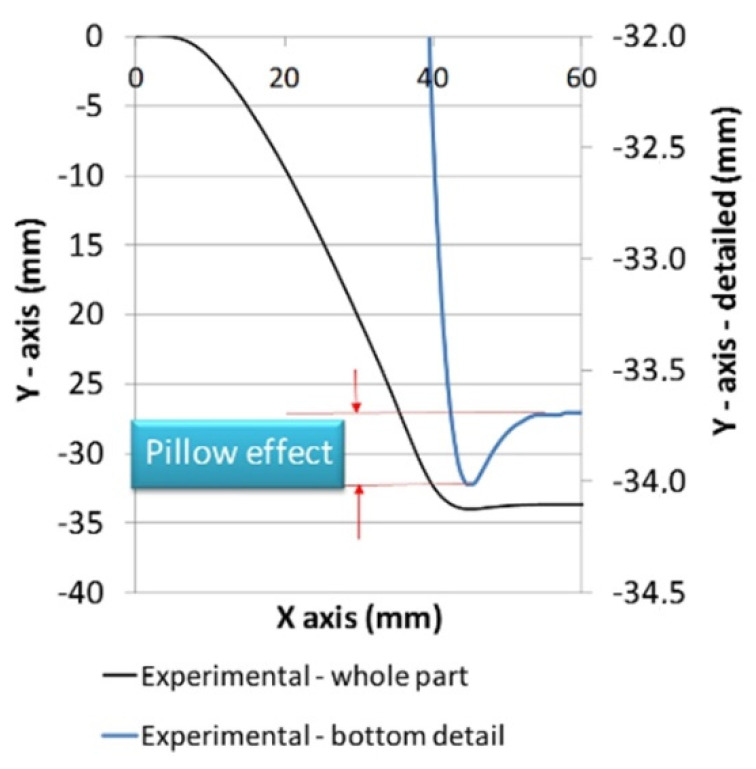
Measured part profile with pillowing detail (one half of the piece only).

The effect of mass scaling size on the reliability of the FE analysis needs to be determined in the first analysis stage. After the experiment and dimensional evaluations of the formed piece were completed, its central area presented in Figure 6 was compared with the results of mass scaling analysis as well as numerical simulations performed with various regimes of tool speed, mass density, and mesh size according to the DoE presented in Table 1. Based on the observed differences, conditions for further work with numerical simulations of SPIF were determined.

When analysing the geometry of the SPIF-produced truncated pyramid, the occurrence of a ‘pillow effect’ (i.e., non-flat workpiece bottom) was observed (Figure 6). This refers to the large elastic springback of material in this zone. The occurrence of the ‘pillow effect’ has an adverse impact on the accuracy of the specimen. This effect is particularly common in the SPIF of large-scale parts. However, the flat bottom being undeformed further emphasises this geometric error. In the selected forming conditions for the experimental truncated pyramid, the pillow effect was detected in the amount of 0.3 mm. Further, profile deviation near the clamped edge due to sheet bending can also be noticed. Neither detected error can be disregarded if narrow tolerances are required.

### 4.1. Effects of Mass Scaling (MS) of the Simulation

**Figure 7:**
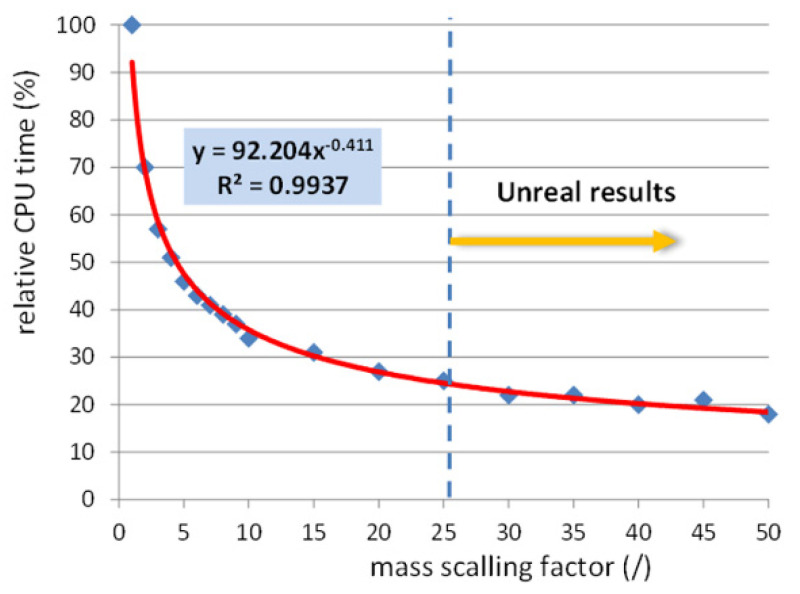
Effects on CPU time by mass scaling of the SPIF process at a punch speed of 40 m/s and element size of 3 × 3 mm.

The analysis of mass scaling was performed on a FE model with other influential parameters fixed: tool speed of 40 m/s and element size of 3 × 3 mm. All simulations aimed to determine the influence of the mass scaling factor on the simulation time necessary to perform FE analysis of the SPIF process were performed under the same hardware conditions. In order to present only the relative change in time spent for the FE simulation, we determined the parameter ‘relative CPU time’ comparing each CPU time at increased mass scaling factors with the reference of MS 1. From Figure 7, it is evident that the time spent for the simulation is significantly shorter with the increase of mass scaling up to the factor of 10, for which the time is only 34% of the reference one. Further increases in MS factor up to the value of 50 show a gradual decrease in time spent for the SPIF analysis. At the MS factor of 25, only 25% of the reference time is necessary, while at the MS factor of 50, the calculation is with relative CPU time of 18% already 5.55 times faster than reference MS factor 1. In Figure 7, the power approximation (power series) with high R^2^ factor of 0.9937 delivers the correlation between the MS factor and relative CPU time used for the SPIF process of the truncated pyramid. Through this, the necessary CPU times at particular MS factors could be calculated. However, it is of highest importance for fast and reliable FE analysis to evaluate the deviations from the reference results being defined from one side with the simulation with the MS of 1 and on the other side with the measured experimental results. Analysis of the pillowing effect in correlation with the mass scaling factor has shown reliable results with minimal deviations in the range below 0.2 mm of the mass scaling up to the value of 10. The range in the MS of 10 to 25 still delivers reliable results of part thickness. However, the geometric error is here increased. Furthermore, at MS factors above 10, a bulge shape is also expressed at central part of the specimen, which is not to be observed in the experimental part.

**Figure 8:**
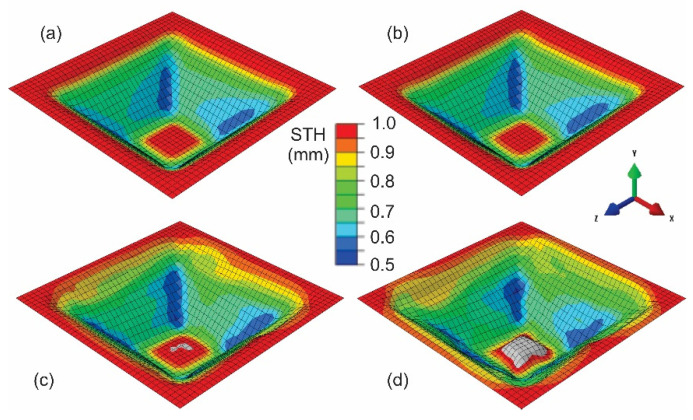
Part thickness at different mass scaling factors of the SPIF process at a punch speed of 40 m/s and element size of 3 × 3 mm—(**a**) mass scale 1, (**b**) mass scale 10, (**c**) mass scale 30, and (**d**) mass scale 50.

Figure 8 presents the simulated part shape and corresponding sheet thicknesses at various mass scaling factors. It is evident that already at the mass scale factor 30, the observed values of part shape show some irregularities. As shown for the MS factor of 50 in Figure 8d, excessive mass scaling delivers significant errors in the observed shape of the specimen. Based on the results given in Figure 7 and Figure 8, it can be concluded that the simulation of the pyramid-shaped test specimen used in the presented study delivered reliable data with the mass scaling factor of up to 25.

### 4.2. Effects of the Mesh Element Size

**Figure 9:**
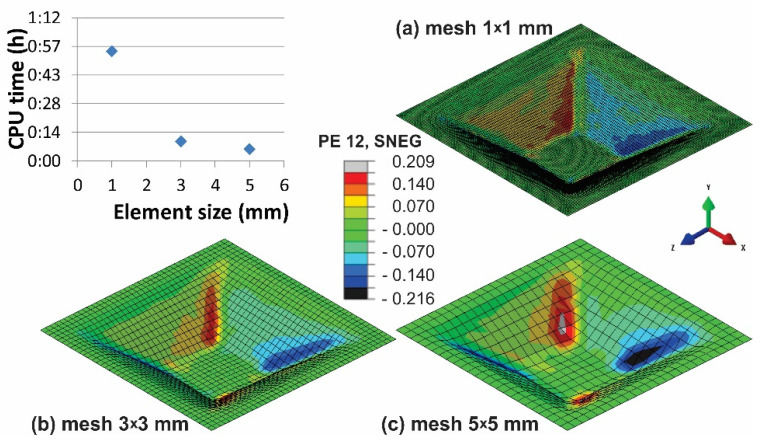
Part strain PE12 at mass scaling factor of 10 after the SPIF process at a punch speed of 40 m/s and element sizes of (**a**) 1 × 1 mm, (**b**) 3 × 3 mm, and (**c**) 5 × 5 mm.

The goal of reliable simulations is to attain the best results in the shortest available time. In the case of the forming processes with long forming times, as in the case of the SPIF process, the proper selection of mass and element size must be evaluated. Once the reasonable combinations of mass scaling and element sizes are selected, their influence on pillowing of the part’s bottom part is to be evaluated. However, the reliability of the obtained results of FE analysis is connected not only with the mass scaling of the dynamic explicit code but also with the selected mesh size. The anomaly of the results from Figure 8 may also be a result of overly coarse selected mesh. In order to evaluate the influence of element size on the accuracy of the performed simulations, a comparative analysis at punch speed of 40 m/s with mass scaling factor of 10 was performed for the mesh sizes 1 × 1 mm, 3 × 3 mm, and 5 × 5 mm. At the same time, the used CPU time for each mesh size was analysed as well. As expected, the CPU time needed to evaluate the model with a mesh size of 1 × 1 mm is drastically increased (Figure 9) in comparison to the mesh size of 3 × 3 mm. In contrast, the reliability of the results is improved as well. From the accuracy point of view; it is evident that the most desired mesh size is 1 × 1 mm, but such a simulation requires 5.43 times more calculation time than the one with the mesh size of 3 × 3 mm and 9.17 times more than the one with a mesh size of 5 × 5 mm. However, the element size of 5 × 5 mm is far too coarse to deliver reliable results. Through this, the combination of the SPIF process parameters for producing the truncated pyramid presented in Table 1 are approved as a reasonable combination for the evaluation of bottom pillowing.

The influential parameters of FE prediction of the SPIF were selected according to the shown separate analyses of mass scaling and FEM element size being incorporated into the design of experiment (DoE) with the parameter ranges of mass scaling of 1 to 25, tool velocity from 10 m/s to 40 m/s, and two mesh sizes of 1 × 1 mm and 3 × 3 mm.

### 4.3. Setup of Design of Experiment and Neural Network Training

**Figure 10:**
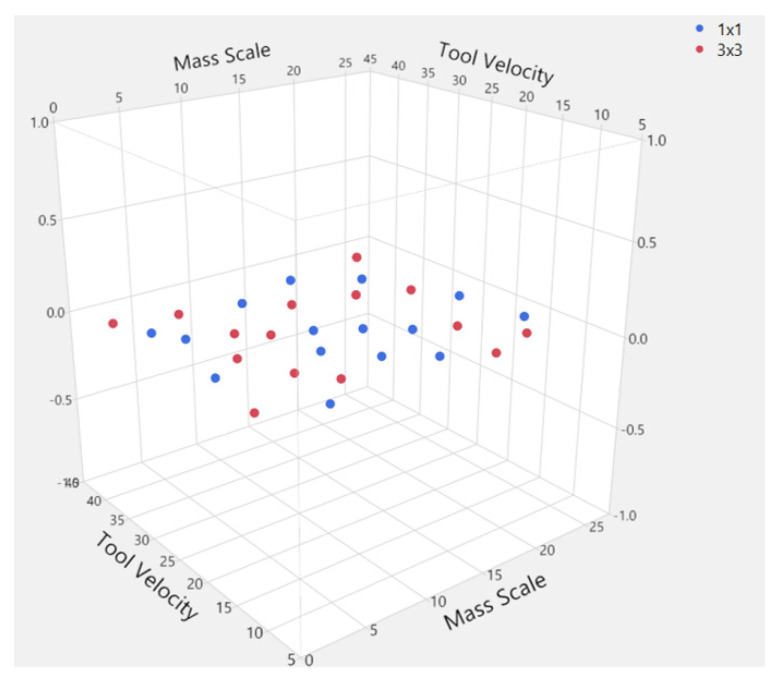
Design points generated by the JMP Fast and Flexible Filling Design.

The range of the most important influential parameters of the mass scale factor, tool velocity, and two ranges of element sizes were combined into a DoE plan as described in Section 3.4. The plan of 30 different combinations of mass scaling, tool velocity, and element size is presented in Table 1, while the design points generated by the JMP Fast and Flexible Filling Design are shown in Figure 10.

To obtain the final model of neural network training, the five models derived for the optimal value of the tuning parameter were fit to the entire data set. The model that had the highest validation log-likelihood was selected as the final model, and the results reported pertain to that model. The relevant measures obtained during training and validation for the three output variables are shown in Table 2, Table 3 and Table 4.

**Figure 11:**
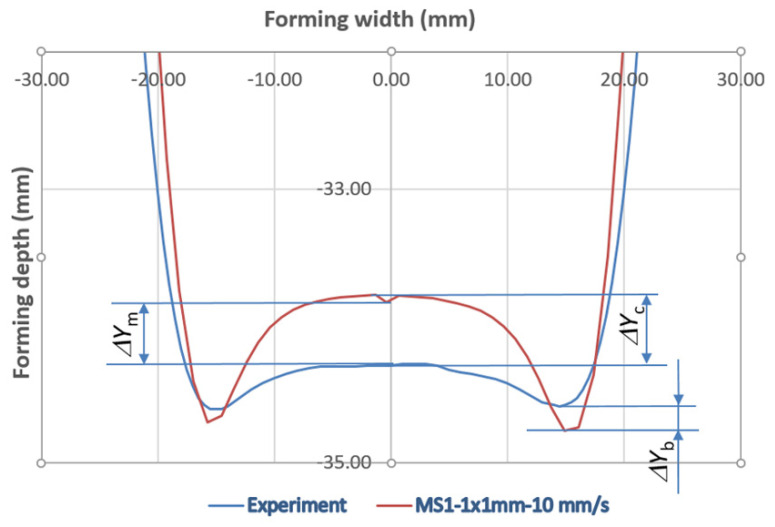
Comparison of experimental results with the results from the reference simulation of the pillowing effect (bottom part of the specimen only).

The deviations of particular simulated shape were compared with the measurements of the SPIF-produced part regarding the size of the arisen pillow defined as the difference between the pillow border *ΔY*_b_ and the pillow near the centre *ΔY*_c_ as marked in Figure 11 for the comparison between experiment and the reference simulation. 

All geometrical data of the pillowing are presented in Table 5. The values of the shifted pillow centre are defined with parameter *ΔY*_m_. From these data, the geometric error due to the central deformation of the part can be calculated. The magnitude of the pillow measured on the experimentally obtained specimen was *ΔY*_p,exp_ = 0.32 mm. Finally, the size of the simulated pillowing, defined as *ΔY*_p,sim_, was calculated for each combination of mass scaling, tool velocity, and mesh size. The *ΔY*_p,exp_ and *ΔY*_p,sim_ are considered as absolute values. The comparison of calculation times needed for the simulation of the SPIF process (excluding the calculation of springback with the standard solver) for the performed combinations of mass scaling, element size, and tool speed is also presented in Table 5. The simulation time needed to calculate the test with MS factor 1, tool velocity 10 m/s, and element size 1 × 1 mm was considered as the reference time. The reference time was 09:16:52 h.

The analysis of the obtained results (Table 5) shows that the influence of the mass scaling observed at a constant forming speed between 10 m/s and 40 m/s does not significantly influence the shape of the simulated pillow in the observed MS factor range from 1 to 25. On the other side, the element size has (as expected) influence on the formed shape of the bottom pillowing. At a larger element size of 3 × 3 mm, less accurate simulation results are observed, while the obtained pillowing is smaller. Furthermore, the spread of the obtained FEM results of the pillowing is here significantly larger than with the 1 × 1 mm element size. However, all simulations express more emphasised bottom pillowing than in the case of the experimentally obtained part. It is evident from Table 5 that the pillow effect is emphasised, and it measures at the largest part 1.07 mm when using a mesh element of a size of 1 × 1 mm. At the same time, the central part of the bottom edge of the workpiece is shifted downwards by 0.1 mm. This effect does not appear on the real part at all. Finally, the central part of the simulated pillowing *ΔY*_c_ differs in drawing depth from the real part from 0.37 mm to 0.57 mm depending on the observed DoE run. In contrast, the outer area of simulated pillowing at the transition zone to the part’s wall *ΔY*_b_ is between 0.12 mm to 0.34 mm larger than measured on the real part.

If a mesh element with a size of 3 × 3 mm is used, the largest observed pillow effect measures 0.80 mm, but the simulated geometry of the bottom of the piece lies mainly below the experimentally obtained shape of the workpiece. In contrast to the results obtained with the mesh size of 1 × 1 mm, the simulated part measured by the parameter *ΔY*_b_ differs in this case from 0.57 mm to 0.87 mm at the outer area of the pillowed bottom. However, the difference between the simulated and real part in its centre is from 0.24 mm to 0.59 mm. The shift of the central point of the specimen is with the 3 × 3 mm element size 0.29 mm, which is three times bigger than in the case of 1 × 1 mm element size. In all cases, the simulations show a slightly larger bend of the workpiece at the tool trajectory.

The size of the mesh element commonly has a quadratic dependence on the CPU time. As the size of the mesh element decreases, the calculation time increases quadratically. For example, under identical conditions, the time needed for simulation calculations for a mesh element with a size of 1 × 1 mm was nine times the time that was needed for the mesh element with a size of 3 × 3 mm. Therefore, a fine mesh is to be applied only in the cases where the prediction of process parameters is crucial for the geometrical accuracy of the formed piece. For general evaluations of proper tool path and part shaping, the mesh could be coarse, and a mass scaling factor up to 25 can be used.

**Figure 12:**
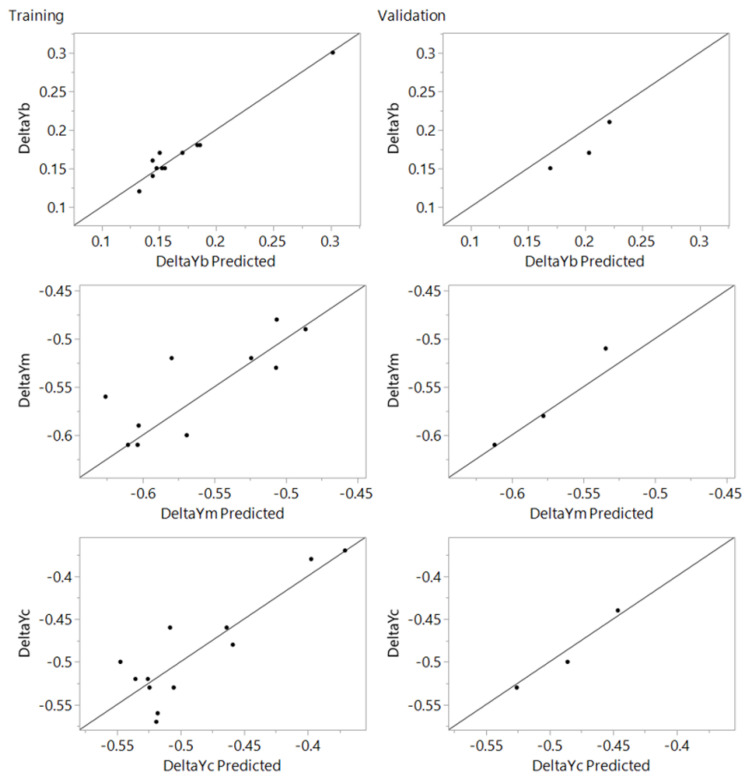
Actual by predicted plot for training and validation for the mesh size of 1 × 1 mm.

**Figure 13:**
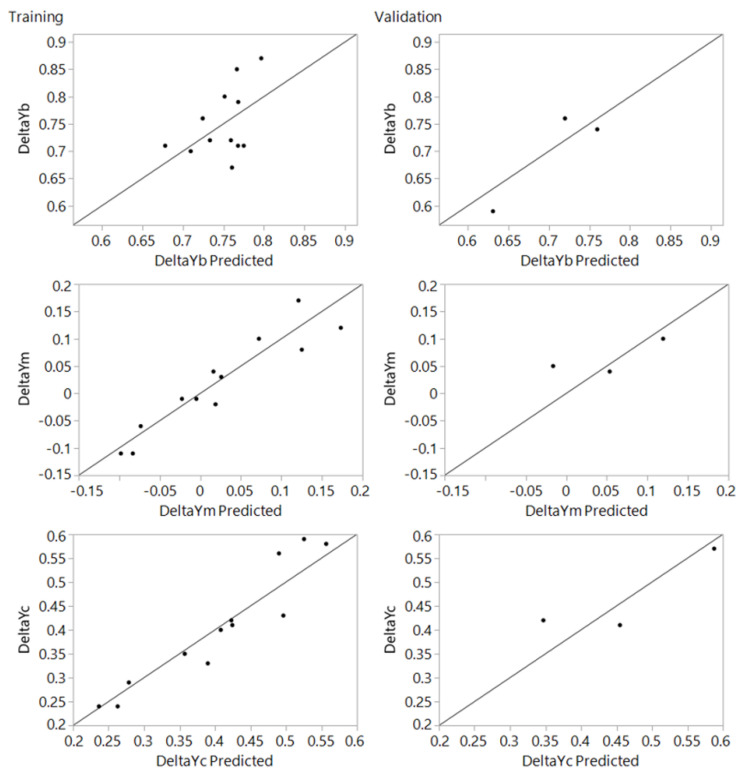
Actual by predicted plot for training and validation for the mesh size of 3 × 3 mm.

**Figure 14:**
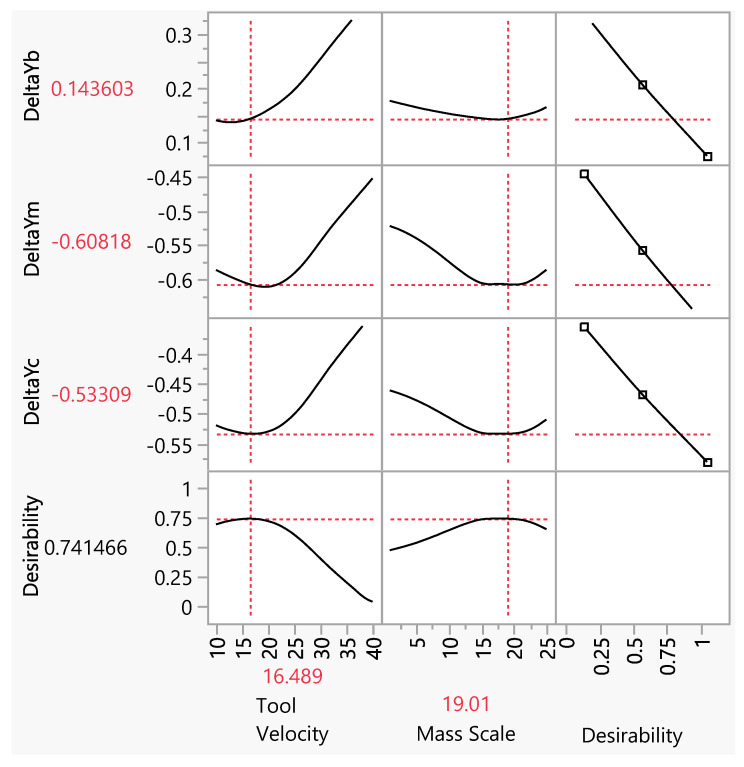
Profiler plot with optimized values for mesh size 1 × 1 mm.

**Figure 15:**
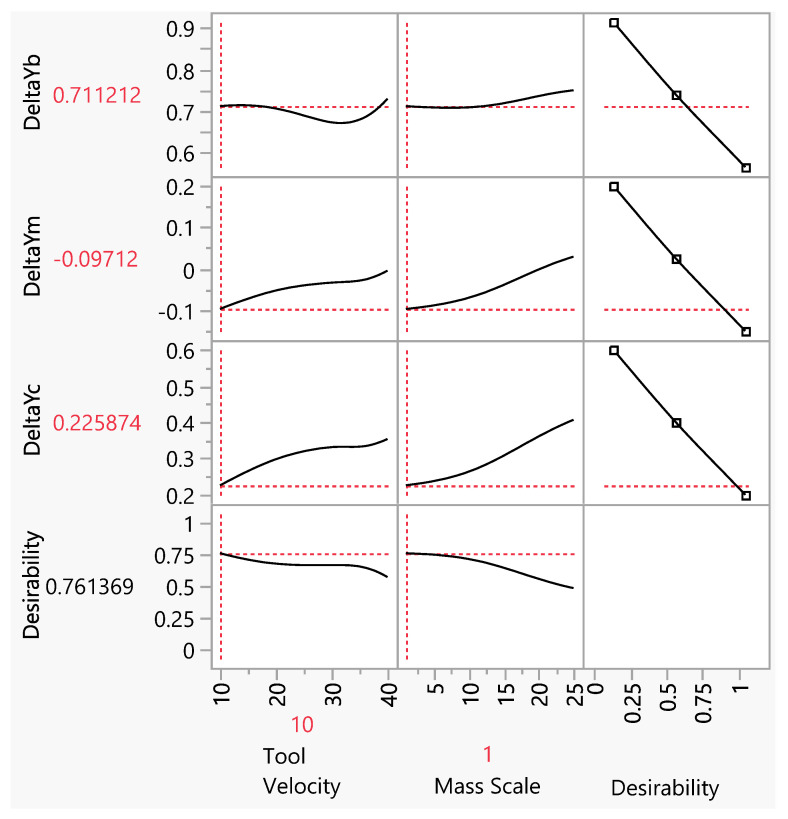
Profiler plot with optimized values for mesh size 3 × 3 mm.

Actual by predicted training and validation plots for the 1 × 1 mm and 3 × 3 mm mesh sizes are given in Figure 12 and Figure 13, respectively, while optimization plots are shown in Figure 14 and Figure 15. From the actual by predicted plots (Figure 12 and Figure 13), it is obvious that the neural network performed better with the data obtained for the 3 × 3 mm mesh size, which is also confirmed by the respective R^2^ values (Table 2, Table 3 and Table 4), which show the percentage of variability that the model is able to explain.

In order to identify the optimal combination of the investigated factors, a multivariate data analysis was performed with the following variables: deviations of the simulated shape in comparison to the real part at its outer bottom area, differences in the formed depth of the part shape in the middle of a pillowed area, the magnitude of the pillow, and the time spent for the numerical simulation.

The optimal parameter combination for the element size of 1 × 1 mm is the mass scale of 19.01 and the tool velocity of 16.49 m/s. As it is evident from the optimisation plot in Figure 14, this parameter combination delivers the values *ΔY*_b_ = 0.12 mm, *ΔY*_m_ = −0.63 mm, and *ΔY*_c_ = −0.56 mm. The FE simulation delivered for the same mass scaling and tool velocity the values of *ΔY*_c_ of −0.51 mm, *ΔY*_m_ of −0.60 mm, and *ΔY*_b_ = 0.16 mm. Both results are sufficiently close together to use the neural network as a reliable tool for further evaluations.

The neural network 3D scatterplot with cluster centres for the 3 × 3 mm mesh size delivered the optimal parameter combinations at the mass scale of 1 and lowest tool velocity of 10 m/s (Figure 15). Here, the selected combination of both minimal values of the input parameters is to be understood as constituting difficult conditions for the neural network evaluation. The pillowing parameters obtained by the neural network optimization are *ΔY*_c_ of 0.13 mm, *ΔY*_m_ of −0.17 mm, and *ΔY*_b_ = 0.65 mm, and the parameters calculated from the numerical FEM model are *ΔY*_c_ of −0.42 mm, *ΔY*_m_ of −0.02 mm, and *ΔY*_b_ = 0.67 mm. Here, the differences among the obtained pillowing results of both approaches are larger than those observed at mesh size of 1 × 1 mm. Furthermore, the spread of the pillowing curves is here larger than in the case of 1 × 1 mm. On the other hand, the calculation times for larger element size are significantly shorter than those observed at the mesh size of 1 × 1 mm.

**Figure 16:**
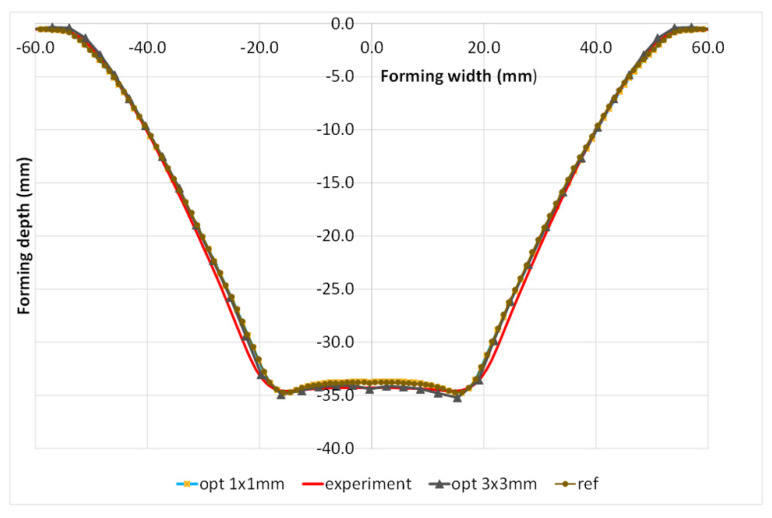
Comparison of experimental results with the FEM results of the DoE-selected parameters of mass scale, tool speed, and element size.

The comparison of the optimal numerical evaluation of the pillowing effect with the experimental one is presented in Figure 16.

## 5. Conclusions

This paper presents numerical analyses and evaluations of commonly used simplifications of numerical simulations for single point incremental forming. The analyses delivered the following findings:

It can be concluded that mass scaling up to 25 times and time scaling up to 40 m/s in numerical simulations can be freely used, since they do not have a crucial effect on the accuracy of the predicted profile, including the pillow effect.

Likewise, the CPU time can be drastically reduced down to 30% of the initial time by implementing the mass scaling effect up to the MS factor of 10. The MS factors between 10 and 25 can be used as well, but some irregularities at fast tool path changes are to be expected. Increasing the size of a mesh element also has an immense effect on shortening the CPU time.

The comparisons between the selected reference simulation having mass scaling factor 1, element size 1 × 1 mm, and tool speed of 10 m/s and simulations with different values of mass scaling, tool speed, and element sizes have shown the decrease in calculation time down to 1%, but the accuracy of such simulation is decreased as well. Therefore, extreme speed-up of numerical simulations has to be used with precaution, since it influences the prediction of the pillow effect.

Finally, analyses of the part bottom deliver excessive values of pillowing in all simulated cases for the analysed case study of a truncated pyramid, which has also been observed by other researchers, and further variable optimisation of the parameters needs to be introduced to decrease this geometrical error.

The performed research work represents a solid base for further use of FEM and ANN methods in shortening the simulation time of incremental forming. This is in particular critical when large-volume parts are processed with incremental forming technology. Therefore, the results obtained here can be beneficiary for the professionals dealing with the design of incrementally formed parts, particularly those with geometrically demanding shapes, for which preliminary FEM verification of the tool trajectories and process parameters are indispensable.

Since the ANN presented here was used in one geometrical case study only, the authors will enlarge the applicability of the presented methods to more complex geometrical shapes as well as various process parameters in order to build an engineering-oriented fast tool for accurate prediction of incremental forming technology.

## Figures and Tables

**Table 1:** Selected design points for ANN training purposes.

Nr. of Run	Mass Scale	Tool Velocity	Mesh Size	Nr. of Run	Mass Scale	Tool Velocity	Mesh Size
1	18.8	10.0	3 × 3	16	9.8	20.7	1 × 1
2	15.5	12.8	1 × 1	17	8.8	27.0	3 × 3
3	24.9	15.1	1 × 1	18	11.6	25.4	1 × 1
4	22.8	11.7	3 × 3	19	2.0	21.8	1 × 1
5	18.2	30.5	3 × 3	20	4.8	24.2	3 × 3
6	14.5	22.7	1 × 1	21	23.4	39.9	3 × 3
7	20.0	17.4	3 × 3	22	20.6	33.9	1 × 1
8	17.1	19.5	1 × 1	23	16.2	38.5	1 × 1
9	24.0	23.5	1 × 1	24	13.3	32.4	3 × 3
10	22.1	28.1	3 × 3	25	1.3	39.4	3 × 3
11	8.2	14.5	3 × 3	26	2.6	34.8	1 × 1
12	12.5	16.2	1 × 1	27	5.9	37.4	3 × 3
13	5.4	10.9	1 × 1	28	10.6	35.9	1 × 1
14	1.1	13.5	3 × 3	29	7.0	29.5	3 × 3
15	6.4	18.2	3 × 3	30	3.8	31.3	1 × 1

**Table 2:** Training and validation measures obtained for *Y*_b_ dependent variable.

Measure	Training		Validation	
	1 × 1 mm	3 × 3 mm	1 × 1 mm	3 × 3 mm
R^2^	0.96333	0.8329518	0.85133	0.9829743
RMSE	0.01215	0.0240331	0.00962	0.0118796
Mean Abs Dev	0.01029	0.0185957	0.00934	0.0116857
Log-Likelihood	−35.89955	−27.71259	−9.67549	−9.041989
SSE	0.00177	0.0069311	0.00027	0.0004234
Sum. Freq.	12	12	3	3

**Table 3:** Training and validation measures obtained for *Y*_m_ dependent variable.

Measure	Training		Validation	
	1 × 1 mm	3 × 3 mm	1 × 1 mm	3 × 3 mm
R^2^	0.76554	0.6807336	0.85159	0.9957846
RMSE	0.02925	0.0464511	0.01614	0.0021425
Mean Abs Dev	0.02146	0.0413569	0.01357	0.0020838
Log-Likelihood	−25.35384	−19.80499	−8.12227	−14.18058
SSE	0.01027	0.0258925	0.00078	1.377 × 10^−5^
Sum. Freq.	12	12	3	3

**Table 4:** Training and validation measures obtained for *Y*_c_ dependent variable.

Measure	Training		Validation	
	1 × 1 mm	3 × 3 mm	1 × 1 mm	3 × 3 mm
R^2^	0.86605	0.7816902	0.95222	0.8552495
RMSE	0.01326	0.0505438	0.00818	0.0296336
Mean Abs Dev	0.00834	0.038575	0.00689	0.0213712
Log-Likelihood	−34.8498	−18.79171	−10.16188	−6.299719
SSE	0.00210	0.0306561	0.00020	0.0026345
Sum. Freq.	12	12	3	3

**Table 5:** Calculation times and geometrical accuracy of simulated model versus the experimental part.

Test No.	Time Factor (%)	*ΔY*_b_ (mm)	*ΔY*_m_ (mm)	*ΔY*_c_ (mm)	*ΔY*_p,exp_ (mm)	*ΔY*_p,sim_ (mm)
Ref. values	100	0.13	−0.46	−0.51	0.32	0.96
1	3.8	0.72	−0.06	0.29		0.75
2	19.5	0.14	−0.56	−0.50		0.96
3	13.6	0.15	−0.61	−0.53		1.00
4	3.1	0.76	0.03	0.40		0.68
5	1.6	0.85	0.17	0.59		0.58
6	11.7	0.18	−0.67	−0.57		1.07
7	1.9	0.71	0.08	0.43		0.60
8	13.1	0.15	−0.61	−0.53		1.00
9	8.9	0.21	−0.51	−0.44		0.97
10	1.3	0.87	0.12	0.58		0.61
11	3.7	0.72	−0.11	0.24		0.80
12	17.2	0.16	−0.61	−0.52		1.01
13	37.4	0.15	−0.53	−0.48		0.94
14	9.0	0.71	−0.11	0.24		0.79
15	3.5	0.76	0.05	0.42		0.66
16	15.3	0.15	−0.59	−0.52		0.99
17	1.9	0.74	0.04	0.41		0.65
18	11.5	0.18	−0.66	−0.56		1.07
19	30.9	0.17	−0.52	−0.46		0.95
20	1.5	0.70	0.04	0.41		0.61
21	1.0	0.59	0.10	0.57		0.34
22	7.0	0.34	−0.49	−0.37		1.04
23	7.3	0.30	−0.48	−0.38		1.00
24	1.6	0.80	0.10	0.56		0.56
25	3.3	0.71	−0.01	0.35		0.68
26	16.0	0.12	−0.60	−0.53		0.97
27	1.8	0.79	−0.01	0.33		0.78
28	9.1	0.17	−0.58	−0.50		0.99
29	2.0	0.67	−0.02	0.42		0.57
30	31.7	0.17	−0.52	−0.46		0.95
